# The Role of Inhaled Chitosan-Based Nanoparticles in Lung Cancer Therapy

**DOI:** 10.3390/pharmaceutics16080969

**Published:** 2024-07-23

**Authors:** Allana Carvalho Silva, Mirsiane Pascoal Costa, Thiago Medeiros Zacaron, Kézia Cristine Barbosa Ferreira, Wilson Rodrigues Braz, Rodrigo Luiz Fabri, Frédéric Jean Georges Frézard, Frederico Pittella, Guilherme Diniz Tavares

**Affiliations:** 1 Postgraduate Program in Pharmaceutical Science, Federal University of Juiz de Fora, Juiz de Fora 36036-900, Minas Gerais, Brazil; allanacarvalho23009@gmail.com (A.C.S.); mirsih.pc@gmail.com (M.P.C.); t.zacaron@gmail.com (T.M.Z.); keziacristine@hotmail.com (K.C.B.F.); wbraz@hotmail.com (W.R.B.); rodrigo.fabri@ufjf.br (R.L.F.); frederico.pittella@ufjf.br (F.P.); 2 Department of Biochemistry, Institute of Biological Sciences, Federal University of Juiz de Fora, Juiz de Fora 36036-900, Minas Gerais, Brazil; 3 Department of Physiology and Biophysics, Institute of Biological Sciences, Universidade Federal de Minas Gerais, Belo Horizonte 31270-901, Minas Gerais, Brazil; frezardf@gmail.com; 4 Department of Pharmaceutical Science, Faculty of Pharmacy, Federal University of Juiz de Fora, Juiz de Fora 36036-900, Minas Gerais, Brazil

**Keywords:** lung cancer, anticancer drugs, siRNA, pulmonary drug delivery, chitosan, nanoparticles

## Abstract

Lung cancer is the leading cause of cancer-related mortality worldwide, largely due to the limited efficacy of anticancer drugs, which is primarily attributed to insufficient doses reaching the lungs. Additionally, patients undergoing treatment experience severe systemic adverse effects due to the distribution of anticancer drugs to non-targeted sites. In light of these challenges, there has been a growing interest in pulmonary administration of drugs for the treatment of lung cancer. This route allows drugs to be delivered directly to the lungs, resulting in high local concentrations that can enhance antitumor efficacy while mitigating systemic toxic effects. However, pulmonary administration poses the challenge of overcoming the mechanical, chemical, and immunological defenses of the respiratory tract that prevent the inhaled drug from properly penetrating the lungs. To overcome these drawbacks, the use of nanoparticles in inhaler formulations may be a promising strategy. Nanoparticles can assist in minimizing drug clearance, increasing penetration into the lung epithelium, and enhancing cellular uptake. They can also facilitate increased drug stability, promote controlled drug release, and delivery to target sites, such as the tumor environment. Among them, chitosan-based nanoparticles demonstrate advantages over other polymeric nanocarriers due to their unique biological properties, including antitumor activity and mucoadhesive capacity. These properties have the potential to enhance the efficacy of the drug when administered via the pulmonary route. In view of the above, this paper provides an overview of the research conducted on the delivery of anticancer drug-loaded chitosan-based nanoparticles incorporated into inhaled drug delivery devices for the treatment of lung cancer. Furthermore, the article addresses the use of emerging technologies, such as siRNA (small interfering RNA), in the context of lung cancer therapy. Particularly, recent studies employing chitosan-based nanoparticles for siRNA delivery via the pulmonary route are described.

## 1. Introduction

Cancer is characterized as a severe public health issue resulting from biological, physical, or chemical insults that induce genetic damage in cells, leading to potential unlimited replication and suppression in the regulation of cellular proliferation [1,2]. Despite notable advances in treatment, the prevalence and mortality rates of this condition remain significantly high on a global scale [3].

It is estimated that approximately 28.4 million new cases of cancer will be diagnosed by 2040 [4,5]. In this regard, breast, prostate, and lung cancers are the most prevalent in both men and women, with the highest incidence and mortality rates associated with lung cancer considering both sexes [6,7,8].

The therapeutic strategy for lung cancer is complex, involving multiple treatment modalities, including surgery, radiotherapy, and chemotherapy, which depend on the stage of malignancy and the patient’s physical state [9,10]. Among the various therapeutic modalities available, chemotherapy represents a principal approach to the prevention of cancer cell proliferation [11]. However, traditional therapies exhibit nonspecific targeting, high rates of severe adverse reactions, low bioavailability, and drug resistance development, all of which limit therapeutic efficacy [12,13,14]. 

In light of these challenges, new strategies have been proposed to overcome them. In this context, the development of drug delivery systems based on nanotechnology has gained prominence in recent decades [15,16]. Nanotechnology represents a highly promising field of research that offers possible approaches to multiple challenges in the diagnosis and treatment of cancer, including lung cancer [10,17]. This technology has the potential to enhance imaging techniques and the delivery of therapeutic agents through effective vectorization to the biological site and optimization of pharmacokinetics and pharmacodynamics, with the aim of reducing toxicity, improving drug stability and solubility [10,12,18,19]. 

The products Abraxane^®^ and Pazenirl^®^ are examples of nanotechnology-based drugs that have been approved by the FDA for clinical use in the treatment of lung cancer. Both are composed of nanostructured paclitaxel, administered systemically [13,20]. However, the administration of this route can be compromised due to low selectivity, with consequent adverse effects, as well as the narrow therapeutic index, which requires careful monitoring to ensure proper administration and minimize potential complications [21]. 

As a result, numerous studies are being conducted with the aim of achieving targeted delivery of nanostructured antitumor drugs directly to the lungs, yielding promising results [22,23,24]. This promising approach offers several advantages, including increased retention in lung tissue, a reduced dose, and mitigation of systemic side effects [25,26,27,28].

Among the nanostructured carriers commonly used in the delivery of therapeutic agents, polymeric nanoparticles of natural origin, specifically made up of chitosan (CS), have been widely explored due to a series of advantages, including biodegradability, biocompatibility, low immunogenicity, and low toxicity [10,11,29,30]. Furthermore, the mucoadhesive property, antitumor and antiangiogenic activities, and the ability to increase drug permeation render CS particularly favorable for pulmonary administration in the context of cancer treatment [31,32]. 

In view of the above, this study focuses on recent research and prospects for the treatment of lung cancer using inhalation formulations made up of CS nanoparticles. The data were collected from the European Patent Office (Espacenet), WIPO (World Intellectual Property Organization), Scopus, ScienceDirect, and PubMed, covering the period from 2004 to 2024.

## 2. Lung Cancer

It is estimated that approximately 1.8 million cases of lung cancer are diagnosed annually, representing approximately 18% of all cancer-related deaths in both sexes [33,34]. This incidence rate is strongly correlated with smoking, which increases the risk of developing the disease by up to 30 times when compared to non-smokers [35]. Other risk factors that contribute to an increased likelihood of developing lung cancer include exposure to substances such as asbestos, arsenic, beryllium, radon, bis-chloromethyl-ether, chloromethyl-methyl-ether, cadmium, lead, asbestos, chromium, non-arsenic insecticides, strong inorganic acids, manganese, nickel, solid fuels (coal and wood), silica, uranium, and ionizing radiation [36,37,38]. Furthermore, it has been demonstrated that chronic inflammatory processes resulting from fibrosis, chronic obstructive pulmonary disease, and tuberculosis are related to the occurrence of lung cancer [39,40,41].

Lung cancer is characterized by non-specific signs and symptoms, which are commonly present in other diseases. These include cough, hemoptysis, chest pain, dyspnea, and weight loss. Additionally, there is a high incidence of asymptomatic cases [42,43,44]. In this context, the diagnosis of the disease occurs mainly in the advanced or metastatic stage, when the effectiveness of treatments is limited, jeopardizing patient survival [33,42].

Although lung cancer can be categorized into different types, the classification most commonly applied histologically is that which segments tumors into non-small-cell carcinomas and small-cell carcinomas [34,45,46].

Small-cell carcinomas account for approximately 15% of cases and are characterized by the presence of central masses with endobronchial growth and a bulky appearance [47]. The inactivation of key suppressor genes involved in regulating cell cycle progression, RB1 and TP53, is frequently associated with clinical diagnosis. Thus, the clinical evolution of small-cell carcinoma is more aggressive, exhibiting high metastatic, invasive, and angiogenic potential [48,49]. The most common sites of metastatic dissemination include the brain, liver, adrenal glands, and bone. Additionally, it is frequently associated with paraneoplastic endocrinopathies [48].

In contrast, non-small-cell carcinomas account for approximately 85% of lung malignancies and are classified into histological subtypes, including lung adenocarcinoma (40–45%), lung squamous cell carcinoma (25–30%), and large-cell lung cancer (5–10%) [14,45]. 

Lung adenocarcinoma, the most prevalent form of primary lung cancer, is distinguished by a high degree of heterogeneity at various levels, including histological, cellular, and molecular. Consequently, genomic instability, encompassing somatic mutations, amplifications, copy number gains, and chromosomal rearrangements, represents a significant defining feature [50]. Genomic studies of human adenocarcinoma biopsies have revealed a number of genetic alterations that are associated with clinical diagnosis, with the *TP53*, *KRAS*, *KEAP1*, *STK11*, *ALK*, and *EGFR* genes standing out [50,51,52]. 

In this way, the classification and understanding of lung cancer defines the anatomical extent and severity of the disease, helping to establish the therapeutic strategy. Chemotherapy is usually used for lung cancer, either alone or in combination with other therapeutic strategies [15].

The Food and Drug Administration has granted the use of more than 300 chemotherapeutic agents for the treatment of cancer. However, due to the heterogeneous nature of these agents, there has been a limitation in the application of these chemotherapeutic agents [30]. The antineoplastic drugs cisplatin, erlotinib, docetaxel, carboplatin, etoposide, and paclitaxel represent the principal therapeutic regimens employed systemically in the treatment of lung cancer (Table 1) [53].

Nevertheless, these drugs are associated with significant cytotoxic effects, including nephrotoxicity, neurotoxicity, pancytopenia, alopecia, gastrointestinal discomfort, compromised fertility, and allergic reactions in the epithelial tissue. Furthermore, systemic administration results in low bioavailability, poor selectivity for the pulmonary environment, and rapid decline in plasma concentration to subtherapeutic levels [76,77].

Thus, the search for new therapeutic approaches has intensified in recent years [78]. In this context, research into the use of RNA interference (RNAi) for the treatment of lung cancer has been promising and has generated significant interest in the scientific community [79,80]. RNAi is a mechanism of gene regulation that can be harnessed to silence specific genes associated with cancer growth and spread. In the context of lung cancer, researchers are exploring RNAi’s ability to target genes involved in uncontrolled cell proliferation, chemotherapy resistance, and metastasis, aiming to reduce disease progression and improve treatment outcomes [81,82,83,84]. This approach offers the prospect of more precise and less toxic therapies, potentially overcoming resistance to conventional treatments, representing a significant hope for lung cancer patients. 

Additional advantages, such as the ability to synthesize nucleic acids, enable the precise, rapid, and cost-effective production of siRNA. This process is relatively low-cost compared to the production of small molecules and antibodies. Furthermore, these advantages contribute to a broader and more versatile therapeutic scope, which significantly drives research into the development of targeted delivery systems [85].

Despite the promising potential of siRNA-based drugs to treat cancer, no such drug has yet received FDA approval [86]. Nevertheless, a considerable number of siRNA-based drugs are currently undergoing clinical trials with the objective of developing new pharmaceutical agents [87]. In the field of lung cancer, Nitto BioPharma initiated a clinical efficacy study of the siRNA drug NBF-006 in January 2019. This study aims to analyze the safety, pharmacokinetics, and preliminary efficacy of intravenous NBF-006 in patients with non-small-cell lung cancer with *KRAS* gene mutations. The preliminary findings from the preclinical studies were encouraging, although the study is still in progress [88,89]. Furthermore, numerous therapeutic targets for siRNA molecules have been identified, and several preclinical studies have demonstrated the efficacy of siRNA-based approaches for the treatment of lung cancer [90,91,92].

Direct administration of free siRNA into the body has a low efficiency rate, as they are rapidly degraded by nucleases after systemic administration, as well as showing non-specificity to the target site and an inability to permeate cell membranes due to their negative charge. Thus, the development of efficient and safe delivery vectors is essential for gene transfection [15,86,93,94]. In the current context, siRNA delivery vectors are classified into two categories: viral and non-viral vectors [15,95]. 

Viral vectors utilize genetically modified viruses that are highly efficacious in nucleic acid transfection. Nevertheless, their utilization may be disadvantageous due to their high cost, intense immunological responses, and oncogenic effects, which restrict their application [96,97,98]. Conversely, non-viral vectors, such as cationic polymers, are regarded as safer and more stable for the delivery of genetic material. They permit greater flexibility, well-defined physicochemical properties, and large-scale preparation, making them preferential choices as gene carriers [99].

Cationic polymers, such as nanoparticles composed of CS, are highly recommended for siRNA delivery due to their additional advantages. The cationic charge of CS can interact electrostatically with the anionic charge of nucleic acids, thereby protecting the genetic material from enzymatic degradation. They also possess several other beneficial properties, including low immunogenicity, sustainability, biocompatibility, endosome escape, mucoadhesiveness, and biodegradability [100]. Furthermore, the positive charge of CS enhances permeability by enabling efficient electrostatic interactions with the negatively charged target cell membrane [34,96].

## 3. Pulmonary Drug Delivery: Perspectives and Challenges

**Figure 1:**
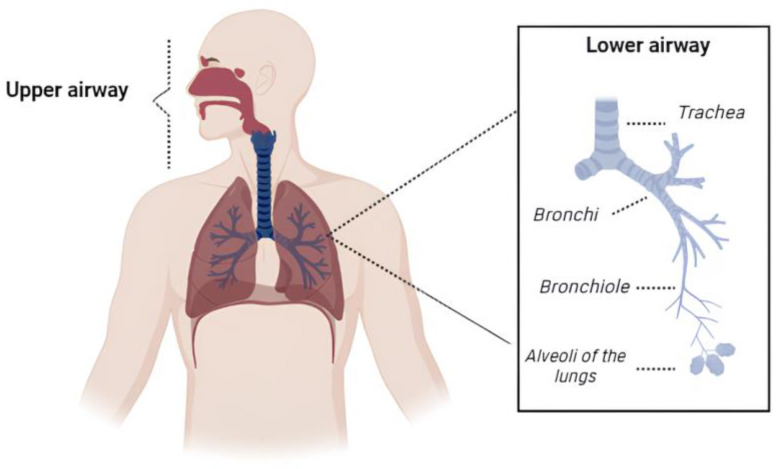
Anatomy of the human respiratory system.

The pulmonary tract is a biological system comprising a series of tissues and organs that are involved in the respiratory process. The upper airways, which are formed by the nasal cavity, larynx, and pharynx, are responsible for conditioning and conducting atmospheric air. In contrast, the lower airways, which include the trachea, bronchi, bronchioles, and pulmonary alveoli, play a fundamental role in gas exchange (Figure 1) [101,102].

The pulmonary region is characterized by providing a large surface area, approximately 100 m^2^, and a thin epithelial layer covering the airways, with a thickness ranging from 0.2 to 1 μm. It exhibits low enzymatic activity and extensive vascularization, factors that favor both local and systemic administration of therapeutic agents via the pulmonary route [103,104]. 

Thus, the potential for reducing therapeutic exposure to healthy cells in other systemic organs, as well as the local administration of reduced doses of the active ingredients to lung tissues and a reduction in adverse effects, represent advantages of administering drugs via the pulmonary route for the treatment of lung cancer [30,105]. Other factors that contribute to the growing interest in pulmonary administration of drugs include the non-invasive nature of the route, which facilitates patient compliance with treatment, and the absence of first-pass metabolism, which overcomes various limitations associated with conventional oral and parenteral therapies [32,106,107,108].

However, the administration of drugs via the pulmonary route presents significant challenges. In order to develop an effective and safe therapy, it is necessary to consider a number of factors, including the cellular target, the type of carrier, particle size, and physiological barrier [103,105].

Particle size, expressed as aerodynamic diameter, is a crucial parameter in the development of pulmonary delivery systems, along with surface charge and morphology [109,110]. The significance lies in the fact that particle size is related to the mechanisms of impaction and distribution in the respiratory tract. Thus, larger particles, with an aerodynamic diameter exceeding 5 µm, are more likely to impact on the airway wall at bifurcations, typically depositing in the upper airways. In contrast, small particles, with an aerodynamic diameter below 1 µm, are exhaled during breathing [53,108,111,112]. Therefore, the ideal particle size to promote efficient deposition in the lower respiratory tract ranges between 1 and 5 µm [95,113]. In this context, nanoparticles would be less effective if administered alone. Therefore, there is a need to develop a suitable drug delivery system based on microparticulate carriers to facilitate the efficient delivery and deposition of nanostructures in the pulmonary environment [22]. 

The morphology and surface charge of particles are of great interest in the bioavailability of therapeutic agents for inhalation. Surface roughness or porous particles reduce interparticle cohesion and contact area, imparting high dispersion [114]. Moreover, pollen-shaped particles exhibit better flowability due to their lower packing density and the presence of conical protrusions that promote spacing between particles, minimizing cohesion and aggregation compared to plate- and needle-shaped particles [105,114].

The surface properties of particles with a high cationic surface charge demonstrate stability and the capacity to permeate lung tissues. However, high charges have been associated with toxic effects [53]. Conversely, the presence of negative surface charges impairs the effective delivery of the therapeutic agent due to charge repulsion with cell membranes, which are also negatively charged. Consequently, in the context of inhalation formulations based on nanoparticles, the nanoparticles can be modified to favor surface alterations aimed at an efficient and safe charge for the delivery of the therapeutic agent to the lungs [53,114].

The innate barrier mechanisms of the lungs are designed to prevent the entry of exogenous particles into the respiratory tract. Therefore, it is important to understand the specific properties of the respiratory system, including the extracellular and intracellular barriers, when developing an inhalation formulation [93].

**Figure 2:**
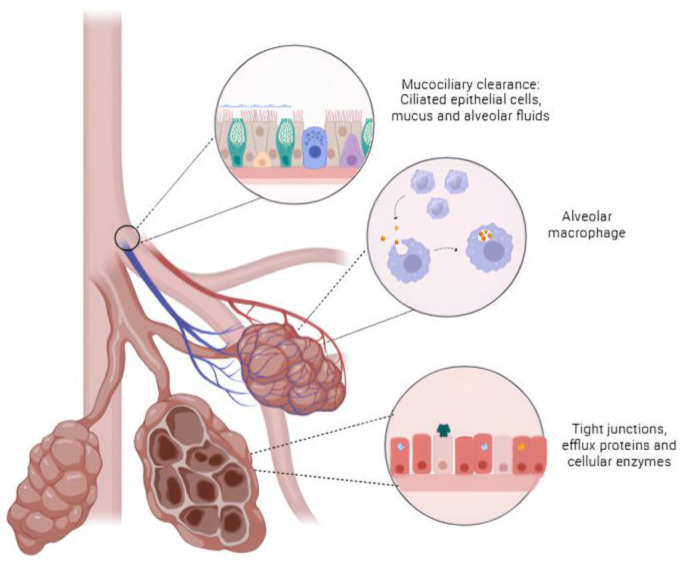
The major physiological barriers involved in the delivery of therapeutic agents to the lungs. The image shows the cellular and non-cellular barriers involved in the process of administering therapeutic agents via the pulmonary route.

One of the primary challenges in pulmonary administration is the presence of ciliated epithelial cells that transport mucus and alveolar fluids, with mucociliary clearance being a vital process responsible for the removal of debris and any unwanted particles inhaled. Another physiological barrier that affects lung delivery is the presence of alveolar macrophages, immune cells designed to phagocytose microorganisms and exogenous particles [107,115]. Furthermore, tight junctions, efflux proteins, and cellular enzymes present in lung tissue act as significant barriers in the absorption process of therapeutic agents [111] (Figure 2). Consequently, both physiological and non-physiological barriers play a significant role in reducing the residence time of drugs in the lung, thereby limiting their delivery to the desired site of action [116].

## 4. CS: A Unique Polymer for Pharmaceutical Applications 

**Figure 3:**
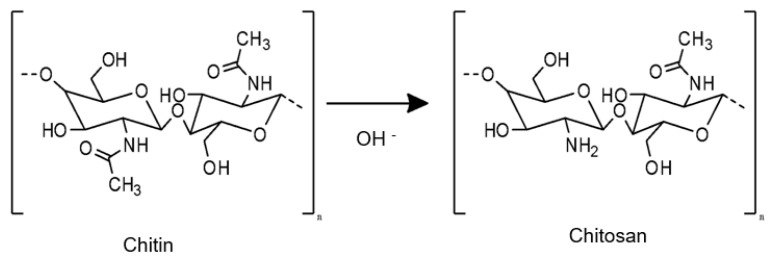
Representation of the chitin deacetylation process for the formation of CS. Chitin is subjected to a treatment process in which it is immersed in a concentrated solution of sodium hydroxide (NaOH) at high temperatures for a prolonged period of time. This treatment process results in the formation of CS as an insoluble by-product.

CS is a linear polysaccharide composed of D-glucosamine and N-acetyl-D-glucosamine units connected by β-(1→4) glycosidic bonds, which is obtained through the deacetylation process of chitin [117,118] (Figure 3). Chitin is a natural biopolymer that is found in the exoskeleton of crustaceans, insect cuticles, and fungal cell walls, ranking as the second most abundant polymer in nature [119,120].

The ratio between the D-glucosamine and N-acetylglucosamine units determines the degree of deacetylation (DD) of the polymer [121]. Despite the absence of a universal consensus on DD values, the majority of commercially available CS exhibits DD values between 60 and 100 percent, with a molecular weight ranging from 3800 to 20,000 Daltons [31].

CS is characterized as a weak base, insoluble in water and organic solvents, but soluble in diluted acidic solutions (pH < 6.5), where the glucosamine units convert to a soluble form, R-NH_3_^+^. Nevertheless, in contact with neutral or alkaline solutions, or in the presence of polyanions, it undergoes precipitation [122,123]. 

CS has attracted increasing attention for pharmaceutical and biomedical applications due to its availability, biocompatibility, biodegradability, low toxicity, and low immunogenicity, as well as its mucoadhesive properties [30,96,108,123,124]. The ability of CS to adhere to mucosal surfaces, such as those found in the respiratory tracts, is attributed to its positively charged amino groups, which interact with the negatively charged mucin glycoproteins present in mucosal secretions [125]. This mucoadhesive behavior enables CS to prolong the residence time of drugs at the site of administration, thereby enhancing drug absorption and bioavailability. Moreover, the mucoadhesive nature of CS enables targeted drug delivery to specific mucosal tissues, offering potential therapeutic benefits in the treatment of various diseases, including lung cancer [34,125]. On the other hand, the positive charge of CS may help in the interaction and cellular uptake of nanoparticles made of this polymer. With this in mind, it has been reported that poly(lactic acid-co-glycolic acid) (PLGA) nanoparticles coated with CS showed a greater capacity for uptake by A549 cells (lung cancer cell line) [126].

Furthermore, CS exhibits various pharmacological activities, including antimicrobial, antifungal, antioxidant, anti-inflammatory, wound healing, anti-diabetic, hypolipidemic, and antitumor effects [127,128,129,130,131,132]. The antitumor mechanism of CS is not fully elucidated. However, studies suggest that it is presumably associated with numerous mechanisms that act on antiangiogenic pathways and elevation of reactive oxygen species [31,133,134]. Furthermore, CS can regulate the expression of proteins associated with apoptosis, activating caspase-9 and caspase-3 cleavage, thus inducing apoptosis through the mitochondrial pathway [11,135]. 

With regard to antitumor activity, the physicochemical properties of CS influence its mechanism of action [31,136]. There is evidence that increasing the DD enhances the in vitro antitumor activity [11]. In this case, with the increase in DD, there is an increase in charge density, which favors interaction with tumor cells, which typically exhibit high negative surface charge [137]. Furthermore, a high DD is conducive to gene silencing, as it optimizes the efficiency of uptake and transfection, thereby circumventing the endosomal compartment [138,139]. 

The versatile properties of CS have prompted interest in the development of nanoparticulate delivery systems for pulmonary administration in the treatment of lung cancer.

## 5. Preparation of CS Nanoparticles

The preparation of CS nanoparticles encompasses a diverse array of methods, each with its own advantages and limitations [140]. Historically, interest in CS nanoparticles has surged significantly due to their unique properties, including biocompatibility, biodegradability, and low toxicity [34,141]. Various methods can be employed for the preparation of CS nanoparticles, including ionic gelation, reverse micelles, emulsification, coacervation, nanoprecipitation, spray-drying, electro-spraying, among others. The selection of the most appropriate method depends on factors such as particle size range, thermal and chemical stability of the drug, ease of scale-up, and toxicity of the final product [140].

The pioneering work in CS nanoparticle preparation dates back to the initial method of covalent crosslinking, which involves the reaction of CS’s amino group with the aldehyde group of a crosslinking agent, such as glutaraldehyde [142]. Although this method has demonstrated the capability to produce nanoparticles with appropriate diameter and size distribution, its usage has become increasingly limited due to concerns regarding the toxicity of glutaraldehyde [104,140]. The quest for safer and more effective alternatives has spurred the advancement of research into novel CS nanoparticle preparation strategies, aimed at high-impact biomedical and pharmaceutical applications.

In this regard, since its development by Calvo et al. (1997), the technique of ionic gelation has emerged as the most widely employed method, owing to its simplicity, low cost, eco-friendliness, and easy scalability [83,143,144]. In this method, an aqueous solution (pH 7–9) of the crosslinking agent, such as sodium tripolyphosphate (TPP), is slowly added dropwise to an acidic aqueous solution (pH 4–6) of CS. Nanoparticles spontaneously form upon mixing these two solutions through inter- and intramolecular interactions between the anionic groups of TPP and the cationic amino groups of CS [144,145].

With regard to pulmonary administration, the utilization of spray-drying for the preparation of CS nanoparticles represents a promising strategy for developing inhalable formulations tailored for efficient pulmonary drug delivery [146]. Spray-drying enables precise control over particle size, morphology, and dispersibility, ensuring optimal aerosolization properties crucial for deep lung deposition [146,147]. Furthermore, this technique facilitates the incorporation of therapeutic agents or bioactive compounds within the CS matrix, enhancing drug stability and bioavailability. The resulting dry powder formulation exhibits excellent physical stability and prolonged shelf life. Furthermore, the scalability and reproducibility of the spray-drying process render it a viable option for large-scale production [147]. Table 2 provides a comprehensive overview of the key characteristics of the principal methods employed for the preparation of CS nanoparticles. 

In addition to the growing interest in the use of CS nanoparticles as drug delivery systems, several studies have shown that this polymer can be used as a coating for different types of nanostructures, such as other polymeric nanoparticles [24] and solid lipid nanoparticles [154]. The presence of CS on the surface of nanoparticles can impart important properties to the delivery system, particularly mucoadhesive capacity and the ability to increase permeability [155]. In addition, CS coating can contribute to the enhancement of the biological activity of the encapsulated drug [24,156]. Furthermore, CS-coated nanoparticles may have greater colloidal stability than uncoated nanoparticles [157].

## 6. CS-Based Nanoparticles for Pulmonary Delivery in Lung Cancer Therapy

CS-based nanoparticles represent a promising approach for pulmonary drug delivery, distinguished by their unique properties and versatility. The mucoadhesive characteristic of CS facilitates adherence to the mucosal surfaces of the respiratory tract, prolonging the residence time of the drug and improving its efficacy [30,102,158]. Furthermore, CS is chemically modifiable, allowing the incorporation of specific targeting agents, which direct the drugs to target cells in the lung tissue, such as tumor cells [34]. Moreover, due to its antitumor property, CS can contribute to increasing therapeutic efficacy [31]. These combined characteristics render CS-based nanoparticles a robust and promising platform for drug delivery in the treatment of lung diseases, including lung cancer.

In general, nanoparticles can be employed for the active or passive release of drugs in the tumor region. In passive release, drug targeting occurs as a result of the enhanced permeability and retention (EPR) effect. The rapid process of angiogenesis in the tumor environment, where blood vessels are produced quickly and imperfectly, results in the formation of fenestrations (with diameters between 100 and 780 nm) between the cells of the capillary epithelium. These fenestrations allow for greater permeation of nanostructures within the tumor region [159]. The literature indicates that nanoparticles with an average diameter between 50 and 150 nanometers can effectively penetrate these larger fenestrations in neovessels, while avoiding the narrower fenestrations in normal tissue endothelial (5 to 10 nanometers) [160,161]. Furthermore, reduced lymphatic drainage at the tumor site facilitates greater accumulation of these carriers, allowing them to remain at the site of action for longer durations [162].

The addition of active targeting ligands on the surface of nanoparticles also enhances their targeting efficiency toward tumor cells. In this instance, nanocarriers are specifically designed to interact with receptors that are overexpressed in cancer cells, enabling targeted delivery of anticancer agents. These nanoparticles typically contain antibodies, peptides, polymers, DNA aptamers, and small molecules on their surface, facilitating targeted detection and uptake by cancer cells [163,164].

Regarding the aforementioned considerations, several studies have been undertaken to prepare and characterize inhaler formulations incorporating antitumor agents encapsulated within CS-based nanoparticles (Table 3).

### 6.1. CS Nanoparticles as an Effective Carrier for Antineoplastic Drugs

As previously stated, the antineoplastic drugs currently available for the treatment of lung cancer present several limitations, including non-specific nature, the need for high doses, drug resistance, and low bioavailability. This can result in serious toxic effects and low selectivity to carcinogenic tissue [10,12]. In this context, the administration of antineoplastic drugs via the pulmonary route can be employed to reduce systemic adverse effects and to enhance therapeutic efficacy [158,170].

In 1993, one of the earliest studies of the pulmonary administration of 5-fluorouracil (5-FU) for the treatment of non-small-cell lung cancer through nebulization was published. Consequently, the authors observed that tumor tissues exhibited higher drug concentrations than healthy lung tissues, accompanied by a satisfactory antitumor response in vivo. Furthermore, that 5-FU concentration in other organs and plasma was extremely low, contributing to the reduction of systemic adverse events [171].

However, the efficacy of inhaled antitumor drugs can be constrained by the branching pattern of the respiratory tract, drug degradation, and physiological barriers. Consequently, nanoparticulate delivery systems have emerged as a promising avenue for optimizing drug penetration and absorption, enhancing stability and facilitating efficient delivery to the tumor region [20]. In this context, CS nanoparticles have been extensively investigated for the pulmonary delivery of antineoplastic drugs.

Paclitaxel (PTX) was first identified at the end of the 1960s and has since been employed extensively as an antitumor agent for the treatment of a range of cancers, including those affecting the ovaries, breasts, prostate, head and neck, and lungs. PTX is distinguished by its low solubility in aqueous media. Consequently, Cremophor is employed as a vehicle in the commercial product, Taxol^®^ [172]. Nevertheless, this vehicle has the potential to alter the pharmacokinetic profile of PTX, which could result in serious hypersensitivity reactions. Consequently, the search for alternative formulations has led to the development of albumin-bound paclitaxel nanoparticles (Abraxane^®^). This product has been approved by the FDA for the treatment of metastatic breast cancer, metastatic pancreatic cancer, and non-small-cell lung cancer [173]. Nevertheless, a number of researchers have devoted themselves to the development of innovative nanostructured delivery systems for PTX with the objective of enhancing the efficacy and safety of the treatment [174]. With regard to lung cancer, a significant proportion of research is directed towards the potential for more effective targeting of PTX to the lung region, through the development of inhalation formulations [172,173].

In a study published in 2017, Liu and colleagues [22] developed nanoparticles composed of CS for the co-administration of paclitaxel (PTX) and quercetin (QUE). The authors justified the use of QUE on the grounds of its intrinsic biological properties, in particular its capacity to inhibit P-glycoprotein (P-gp), which is responsible for resistance to multiple drugs. The nanoparticles (CNPs) were prepared by conjugating CS and oleic acid and had an average diameter of 226 nm, a polydispersity index (PdI) of 0.123, and a zeta potential (ZP) of +32.0 mV. The encapsulation efficiency (EE) was 92.6% and 90.3% for PTX and QUE, respectively. Subsequently, the nanoparticles were dried by spray-drying in the presence of β-cyclodextrin, lactose, and mannitol. As a result, microparticles containing CNPs with suitable diameter for pulmonary administration (1–5 µm) were obtained, which, upon dilution in water, facilitated easy redispersion of the nanoparticles. In this case, there was a decrease in the mean diameter of CNPs (199.5 nm), while maintaining a narrow size dispersion (PdI = 0.201). These results were corroborated by transmission electron microscopy, which indicated that the microparticles could readily redisperse into CNPs in the polar environment of the alveoli. In the in vitro release test, it was observed that the microparticles containing CNPs were able to sustain the release of the encapsulated drugs in the different dissolution media (pH 4.5 and 7.4). However, a greater quantity of the drugs was released at the more acidic pH. The researchers posit that this phenomenon is due to the greater swelling of CS in an acidic environment. They further suggest that this behavior could ensure the release of greater quantities of the drugs in the tumor region, where the pH is lower than that of the physiological environment. Finally, biodistribution studies indicated that microparticles containing CS CNPs, following pulmonary administration in Wistar rats, exhibited a markedly higher accumulation in the lung when compared to other organs (heart, liver, spleen, and kidneys). 

Resveratrol (RVT) is a polyphenol that has been extensively studied as a potential antineoplastic agent due to its ability to inhibit NF-κB activation, exhibit antioxidant activity, and interfere with cell proliferation via signaling pathways including PI3K/Akt, mTOR, MAPK, and Wnt/β-catenin. Although numerous studies have demonstrated the potential of RVT in cancer treatment, its characteristic low solubility represents a significant obstacle that must be overcome to enhance its bioavailability. The development of effective release systems capable of overcoming this challenge is therefore essential. In light of these considerations, the encapsulation of RVT in nanostructured systems has been extensively investigated [175].

**Figure 4:**
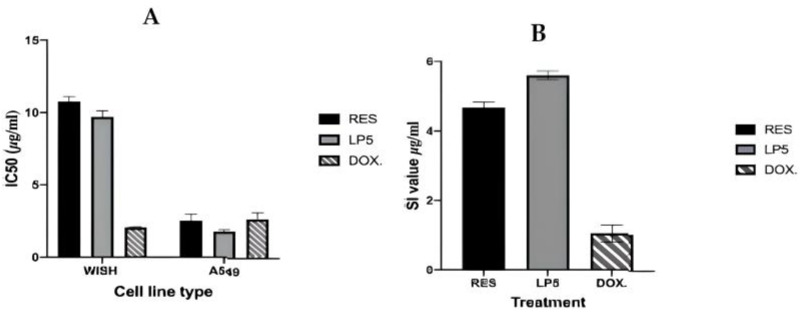
(**A**) The IC50 values in the WISH and A549 cell lines demonstrated that the LP5 formulation significantly amplified the anticancer effects of RES by 63.2-fold. (**B**) In addition, LP5 treatment demonstrated higher anticancer selectivity index values (5.511) in A549 cells in comparison to those of doxorubicin (0.878). Reproduced with permission from [23]; published by Elsevier, 2022.

In this sense, Kamel et al. [23] developed nanoparticles based on lecithin and CS for the encapsulation of RVT for administration by nebulization for the treatment of lung cancer. The nanoparticles exhibited an average diameter of 59 nm, a highly positive ZP, a high encapsulation efficiency (89%), and promising mucoadhesive properties. The nanoparticles were aerosolized in a jet nebulizer, and the data obtained in a cascade impactor enabled the parameters of the fine particle dose (FPD = 2764.52 ± 352.86 μg), fine particle fraction (FPF = 69.41% ± 2.85%), and the mass median aerodynamic diameter (MMAD = 1.37 ± 0.07 μm) to be determined. The authors posited that, when considered collectively, these data may suggest satisfactory delivery of the nanoparticles to the lungs. Moreover, the IC50 values for the nanoparticles (1.7 µg/mL) in A549 cells were found to be lower than those obtained for unencapsulated RVT (2.3 µg/mL) and doxorubicin (2.2 µg/mL) (Figure 4). Furthermore, the nanoparticles exhibited a higher selectivity index (5.51) in comparison to doxorubicin (0.878).

Silibinin (SLB) is a polyphenolic extracted from *Silybum marianum* that has been widely used as an anticancer agent. However, the bioavailability of silibinin is low, necessitating a high dose to achieve clinical efficacy, which can result in systemic toxicity. In this context, Raval et al. [156] characterized and evaluated PLGA/PCL nanoparticles coated with CS containing silibinin for pulmonary administration in the treatment of lung cancer. Following coating, the nanoparticles exhibited an average diameter of 230 ± 1.02 nm, monodisperse characteristics (PdI = 0.35 ± 0.65), a positive ZP (+20.8 ± 1.02 mV), an EE of 65.5 ± 1.08%, and a sustained release of SLB for 48 h. In the evaluation of the in vitro efficacy of the SLB, the authors observed that nanostructuring increased the inhibitory activity of SLB against the A549 strain. The inhalation powder was obtained by freeze-drying, mixed with inhalation-grade anhydrous lactose and characterized in terms of its flow properties and in vitro pulmonary deposition profile. As a result, the authors confirmed that the powder was free-flowing (angle of repose = 39.21°, Carr’s index = 18.11% and Hausner’s ratio = 2.01) and concluded, from the cascade impactor test, that the formulation may be able to penetrate deeply into the lung (MMAD = 4.7 ± 1.8 μm, FPF = 80.2 ± 3.5%). The in vivo pharmacokinetic evaluation was conducted on Sprague–Dawley rats, and the principal findings were that the nanostructured SLB exhibited a notable increase in mean residence time compared to the drug in solution. Moreover, the nanostructuring process resulted in a notable enhancement in the bioavailability of SLB. The authors postulated that the enhancement in pharmacokinetic parameters may be attributed to the mucoadhesive property of CS, which facilitates the prolonged retention of nanoparticles within the lungs. 

2-Methoxyestradiol (2-ME) is a natural metabolite of estrogen with proven anticancer properties for various types of solid tumors due to its antiangiogenic and antiproliferative activities. Nevertheless, the low aqueous solubility and the short half-life of 2-ME limit its bioavailability, thereby jeopardizing its efficacy [176].

Guo et al. [24] developed microparticles containing PLGA nanoparticles coated with CS for the delivery of 2-ME to lung tissue. The authors successfully produced nanoparticles with a mean diameter of 221.2 ± 8.2 nm, ZP of 24.29 ± 0.26 mV, and an EE of 65.34 ± 2.81%. The inhalation powder was obtained via spray-drying, and lactose, leucine, and Poloxamer 188 (5:1:0.028, *w*/*w*) were employed as adjuvants. Following reconstitution of the powder, there was no discernible increase in the diameter of the nanoparticles (mean diameter after reconstitution = 225.7 ± 9.5 nm). Furthermore, the FPF for the inhalation formulation was 55.54 ± 3.11%, a value that is considered adequate for pulmonary deposition of the particles. In the in vitro release test, the authors demonstrated sustained release of 2-ME, with 70% of the drug released over two days. The cytotoxicity of the drug against lung cancer cell lines (SPC-A1 and A549) was higher for nanoparticles coated with CS. This result was linked to the greater cellular uptake of the nanoparticles decorated with CS than the uncoated ones. In fact, there are reports in the literature that CS was able to increase the uptake of PLGA nanoparticles in different cell lines [177,178]. Following intratracheal administration, 2-ME CNP was deposited deeply in the lungs of rats, exhibiting no apparent signs of inflammation, even at the highest dose (10 mg). 

Abba and co-authors [165] investigated the potential of an innovative platform for pulmonary delivery for the treatment of non-small-cell lung cancer. In this study, microparticles based on polyvinylpyrrolidone (PVP) and maltodextrin embedded with CS nanoparticles were obtained by spray-drying and characterized in vitro from a physicochemical, morphological and biological point of view. The authors investigated the properties and performance of CS nanoparticles containing a model drug (fluorescein) (CS NPs) and theranostic CS nanoparticles containing magnetic nanoparticles (CS MNPs). In addition, both nanoparticle formulations were functionalized with an antibody against the epidermal growth factor receptor (EGFR) to enhance tumor cell targeting. The CS-NPS had a mean diameter of 63.0 ± 3.0 nm and a ZP of +28.6 ± 0.6 mV. On the other hand, the mean diameter (104.3 ± 9.9 nm) and ZP (+40.8 ± 0.2 mV) of the CS MNPs were significantly higher. Microscopic images showed that the MNPs (mean diameter = 27.4 ± 2.8 nm; ZP = −26.6 ± 4.6 mV) were uniformly distributed within the CS nanoparticles. The NPs demonstrated increased cellular uptake in A549 cells and the ability to provide sustained release of the model drug (CS NPs). Furthermore, CS MNPs were shown to release the drug in response to an external magnetic field. The microparticles containing CS NPs or CS MNPs had a spherical shape and MMADs of 5.1 µm and 6.1 µm, respectively. It is important to note that for the microparticles embedded with CS MNPs, the mean aerodynamic diameter was above the range recommended in the literature to ensure adequate lung deposition (1–5 µm) [95]. However, for both inhalation formulations, the FPF was ~40%, which is the fraction of particles capable of reaching deep into the lung.

### 6.2. CS Nanoparticles as an Effective Carrier for siRNA

In cancer treatment, the use of the RNAi effect through siRNA molecules aims to silence key genes in tumor progression, thereby reducing the production of proteins responsible for uncontrolled cell growth [179]. siRNA is a double-stranded RNA molecule of 21 to 23 nucleotides that acts at the post-transcriptional level by inducing gene silencing through specific cleavage of the complementary mRNA sequence, thereby inhibiting protein synthesis [180].

Consequently, the genes of choice are those involved in the survival, proliferation and metastatic process of cancer cells [85,181,182]. Among the genes, one of the most extensively studied is that of vascular endothelial growth factor (VEGF), which is responsible for the abnormal growth of blood vessels in tumor tissue. In the context of lung cancer, studies have demonstrated that an siRNA targeting the VEGF gene was capable of inducing near-complete tumor regression in mice [183]. Another oncogene that has been extensively studied in the context of lung cancer is KRAS. The mutation is directly related to the excessive proliferation of cells. Some authors have demonstrated a reduction in cell proliferation and an increase in apoptosis when a siRNA was employed to silence the gene expression of mutated KRAS [184,185,186]. In numerous types of cancer, increased glucose transport into cells, due to exacerbated expression of the transporter, is directly correlated with tumor progression. In a recent study, Li and colleagues [187] demonstrated that the application of siRNA targeting the glucose transporter-1 in lung cancer cells not only inhibited the proliferation of these cells but also induced apoptosis.

βIII-tubulin, a constituent of the microtubules essential for cell division, and Polo-Like Kinase 1 (PLK1), a pivotal enzyme in cell cycle regulation and DNA damage response, are overexpressed in lung cancer cells, promoting tumor growth. siRNAs targeting these genes have demonstrated efficacy in vitro and in vivo, inhibiting tumor growth in mouse models [188]. Sirtuins, acting as tumor suppressors, include sirtuin 6 (SIRT6), which is overexpressed in NSCLC cell lines. Silencing SIRT6 via siRNA induces cell cycle arrest and apoptosis in cancer cells [189]. Similarly, UBA6-specific E2 conjugation enzyme 1 (USE1), another overexpressed enzyme in lung cancer, shows reduced tumor growth upon siRNA-mediated silencing, through cell cycle arrest and apoptosis induction [190].

PD-L1 (Programmed Death-Ligand 1), a transmembrane protein aiding immune evasion, binds to the PD-1 receptor on T lymphocytes, allowing cancer cells to escape immune surveillance. Han et al. [191] employed siRNA to silence PD-L1 expression in both membrane and cytoplasm of NSCLC cells. This direct silencing induced programmed cell death in NSCLC H460 cells independently of T cell involvement, with significant reductions in PD-L1 expression and tumor growth inhibition observed in mouse models. Collectively, these studies underscore the potential of RNAi-based therapies to target diverse genes crucial for lung cancer cell survival and progression.

Nevertheless, the intrinsic instability of the siRNA molecule represents a significant challenge in its therapeutic application. The rapid enzymatic degradation and short plasma half-life of the siRNA molecule limit its clinical efficacy. Furthermore, the negative charge of the siRNA molecule impedes its penetration of target cells [192,193]. Therefore, the need to develop nanostructured delivery systems to stabilize and specifically direct siRNA to target cells is evident. These nanostructured systems not only protect siRNA from enzymatic degradation and early elimination but also facilitate its cellular internalization, thus improving therapeutic efficacy in various applications, especially cancer therapy [194,195].

**Figure 5:**
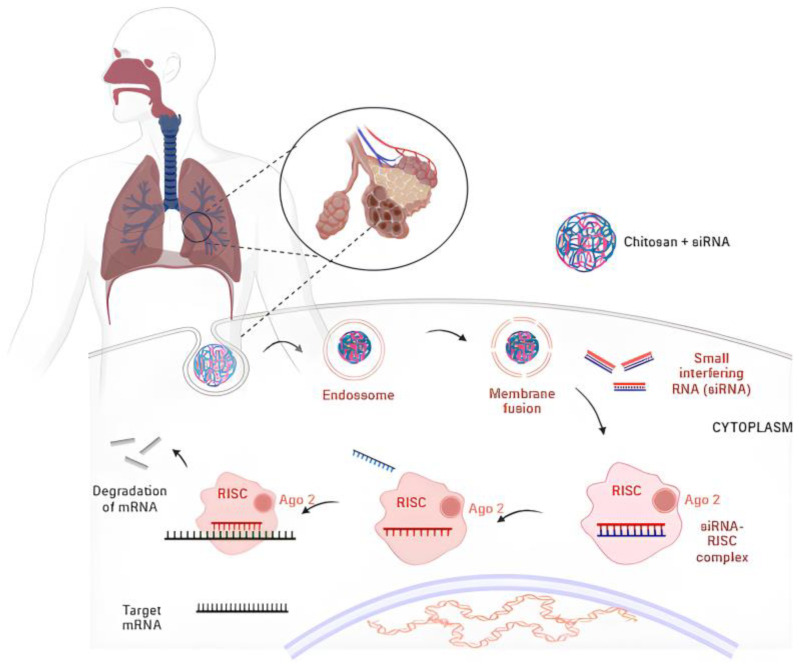
The schematic illustrates the steps involved in the cellular uptake and delivery of siRNA into the lungs. In order for the nanoparticles to be released into the cytoplasm of lung cells, they must escape from the endocytic vesicle. Consequently, during the process of endocytosis, the nanoparticles are localized within the endosome. As the endosome matures, the acidification process occurs, resulting in a pH of 5–6. During the acidification of the endosomes, the primary amines present in CS are progressively protonated, resulting in an influx of chloride ions into the endosomes to maintain charge neutrality and increase the ionic strength in the endosomes. This process results in osmotic swelling and the physical rupture of the endosome, thereby releasing the siRNA into the cytoplasm. Subsequently, the siRNAs associate with the RNA-induced silencing complex (RISC), a large protein complex comprising Argonaut (Ago2) proteins. When bound to RISC, the siRNA unwinds and the sense strand is removed and degraded by nucleases. The antisense strand of the siRNA directs RISC to the target mRNA. The cleavage site is aligned with the Ago2 endonuclease domain, which facilitates cleavage of the phosphodiester bond in the mRNA and the subsequent release of the cleaved mRNA fragments, which are then degraded, resulting in gene silencing.

One of the significant benefits of CS nanoparticles for siRNA delivery is the simplicity of the process involved in obtaining the nanoparticles. In this instance, the negatively charged siRNA can bind to the protonated cationic amino group within the structure of the CS nanoparticles through electrostatic interaction, thus facilitating the formation of stable polyplexes [11,196]. Figure 5 depicts the mechanism of siRNA release from CS nanoparticles and its subsequent mechanism of action [99].

**Figure 6:**
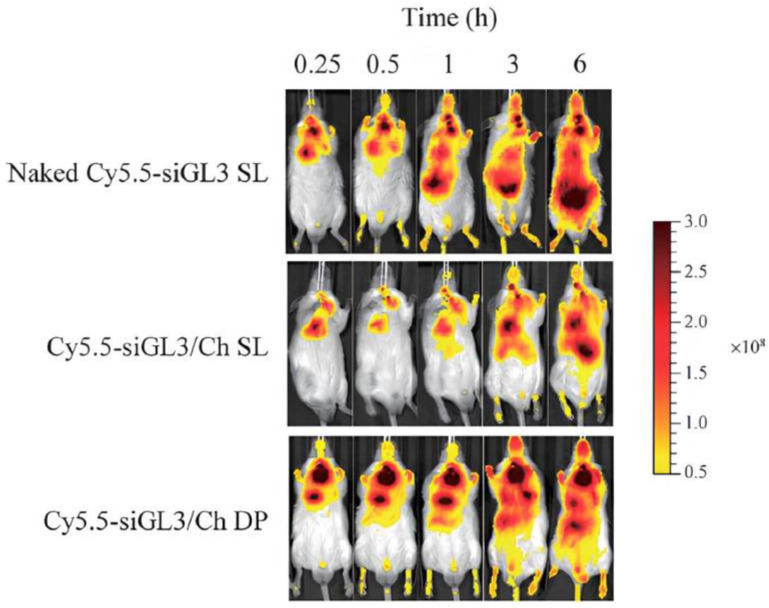
The biodistribution test demonstrated that pulmonary administration of non-encapsulated siRNA led to rapid dispersion from the lung tissue into the systemic circulation. Conversely, the translocation of siRNA from the lung to the systemic circulation and liver was delayed when both the Cy5.5-siRNA/chitosan solution (SL) and the Cy5.5-siRNA/chitosan powder (DP) were administered. This indicates that chitosan prolonged siRNA retention in the lungs. Reproduced with permission from [166]; published by J-STAGE, 2013.

The feasibility of using CS to deliver siRNA to the lungs was demonstrated by Okuda et al. [166], who developed a dry powder formulation containing CS-mannitol nanoparticles and siRNA using the supercritical CO_2_ technique. The objective of the study was to administer siRNA-luciferase in order to evaluate the prolonged exposure of the siRNA/CS complex on the epithelial surface of the lung. The authors observed that the average diameter and ZP of the nanoparticles remained unaltered following the powdering process and demonstrated that the integrity of the siRNA was maintained throughout the supercritical CO_2_ process. Furthermore, the results demonstrated that the formulation significantly inhibited the increase in luminescence intensity in the lungs, indicating effective and specific in vivo gene silencing for metastatic tumor cells in the lungs of mice. The biodistribution test demonstrated that pulmonary administration of non-encapsulated siRNA led to rapid dispersion in lung tissue and systemic circulation, with subsequent detection in the liver and intestine. In contrast, pulmonary administration of CS-siRNA nanoparticles was found to prolong siRNA retention in the lungs, suggesting that CS, due to its bioadhesive properties, may have contributed to the longer pulmonary residence time (Figure 6).

Nielsen and colleagues [167] developed an aerosolized delivery system containing siRNA-EGFP associated with CS nanoparticles for intranasal and intratracheal administration for the treatment of lung cancer. The average diameter (298 nm) and PdI (0.3) of the nanoparticles remained unchanged following nebulization (272 nm and 0.26, respectively). Conversely, the nanoparticles showed a narrower size distribution after aerosolization. Similarly, aerosolization did not affect the capacity to silence the endogenous EGFP in human lung carcinoma cells (H1299) (68% reduction before and 62% after the process). In a murine model, intratracheal administration of nanoparticles resulted in deposition in the apical region of upper and lower airway cells, with a greater degree of gene silencing observed compared to free siRNA and the untreated group. The nanoparticles developed were considered promising carriers for gene silencing in respiratory diseases, specifically for lung cancer.

In a similar vein, Howard et al. [168] developed CS nanoparticles for pulmonary delivery of siRNA-EGFP for lung cancer therapy. The nanoparticles exhibited an average diameter of less than 350 nm and a positive ZP, indicating an excess of CS in the formation of the complex. Fluorescence microscopy demonstrated the intracellular trafficking of nanoparticles containing Cy5-labeled siRNA, with fluorescence observed in the apical regions and cytoplasm of H1299 cells. Furthermore, the researchers observed that following intranasal administration of the nanoparticles in transgenic mice expressing EGFP, there was a significant reduction in EGFP expression in the lower and upper regions of the lung tissue compared to the control group.

Jin et al. [155] developed a CS aerosol formulation containing anti-programmed cell death protein (aPD-L1) with the objective of inhibiting lung cancer metastasis. The average diameter of the nanoparticles was 60 nm, with ZP of +23.8 mV. The results demonstrated the potential of CS as a carrier due to its capacity for absorption and transmucosal penetration, which facilitates the delivery of aPD-L1 to cancer cells. Furthermore, consecutive inhalation was able to activate the immune system, promoting the infiltration of various immune cells, particularly CD8+ T cells, around the tumor, resulting in the apoptosis of cancer cells and prolonging the survival period of the animals.

The phenomenon of multiple drug resistance (MDR) represents a significant obstacle to the efficacy of conventional chemotherapy drugs, thereby jeopardizing the success of cancer therapy [197,198]. In the treatment of lung cancer, several factors are responsible for MDR, including: alterations in the influx and efflux of drugs, alteration or inactivation of the pharmacological target, compartmentalization, epigenetic alterations and DNA damage, blocking of cell cycle arrest and apoptosis, interaction of tumor microenvironments, and metastasis [199].

Consequently, one of the principal strategies for regulating MDR is currently linked to the elevation of antineoplastic drug concentrations, which may result in significant adverse effects in patients [200,201]. Systems based on CS nanoparticles have been investigated for the co-delivery of siRNA and chemotherapeutic drugs, with the aim of enabling the siRNA to selectively silence the MDR-related gene and for the administered antineoplastic to exert their antitumor effects. Furthermore, the synergistic effect can promote a reduction in the dose of the drug, thus preventing undesirable side effects [202]. 

In this context, Xu et al. [169] developed inhalable microparticles containing CS nanoparticles and poly-L-lactic acid (PLLA) for the co-delivery of doxorubicin and siRNA-MRP-1, with the objective of suppressing drug resistance in the treatment of lung cancer and enhancing therapeutic efficacy. CS nanoparticles containing siRNA (siRNA-CS) were prepared using the ionic gelation technique in the presence of TPP. As a result, nanostructures with a spherical morphology and an average diameter of approximately 100 nm were obtained. The ZP of the siRNA-CS nanoparticles was found to be −21.3 mV, which was lower than that observed for the placebo nanoparticles (+34.6 mV). This result was interpreted by the authors as an indication of the successful formation of the siRNA-containing nanoparticles. Furthermore, the nanoparticles exhibited sustained release of the siRNA in a simulated physiological buffer, with 60% release observed within 24 h. Subsequently, the researchers developed porous PLLA-based inhalation microparticles containing the siRNA-CS and doxorubicin hydrochloride (DOX) by the supercritical anti-solvent precipitation process. The resulting microparticles exhibited satisfactory aerodynamic characteristics for effective deposition of the particles in the airways, with an average aerodynamic diameter of approximately 4 μm and a FFP value exceeding 50%. In the in vitro antitumor test, microparticles made up of the combination of siRNA-CS and DOX demonstrated a greater impact on the viability of drug-resistant cells (H69AR cell line, human small-cell lung cancer) when compared to free DOX and microparticles containing only DOX. The authors posit that this finding is attributable to the presence of nanoparticles containing siRNA, which effectively silenced the MDR gene, thereby facilitating the action of DOX.

## 7. Patent Review

The development and exploitation of new nanotechnology-based therapies aimed at overcoming the shortcomings of conventional chemotherapeutic agents is currently underway. In this context, in order to identify patents related to the use of CS in the pulmonary administration of lung cancer treatment, patents obtained from the Espacenet and WIPO platforms over the last 20 years were retrieved. The following combination of keywords was included in the search: “chitosan nanoparticles” and “lung cancer” and “inhaled”. 

No patents were found in the WIPO database with the keywords used. A search of the Espacenet database yielded 37 results. The patents were analyzed individually in order to ascertain the correlation between the use of SC nanoparticles and the purpose of the review. Table 4 presents a summary of patents related to the development of CS nanoparticles for pulmonary administration in the treatment of lung cancer. 

As shown in the table above, only 11 patents were suitable for the purpose of the review. Of these, 36.3% were from China, 36.3% from the United States of America, 9.1% from Turkey, 9.1% from Canada, and 9.1% from India.

Among the compositions obtained in the patents, CS exhibits several favorable properties for pulmonary delivery, including bioadhesive properties to the mucosa, sustained drug release, a favorable safety profile in pulmonary delivery, protection of genetic material during release, and increased bioavailability of the encapsulated agent [172,173,174,175,176,177,178,179,180,181].

## 8. Conclusions and Perspectives

Despite advances in the pharmaceutical industry in developing safe and effective drugs, the global prevalence and mortality rate of lung cancer remain alarmingly high. In the context of nanotechnology, only Abraxane^®^ and Pazenir^®^ have been clinically approved for the treatment of non-small-cell lung cancer via parenteral administration. However, intravenous administration compromises targeted delivery to the desired region and promotes systemic adverse events, potentially limiting therapeutic efficacy. Consequently, the development of drug delivery systems for pulmonary administration represents a promising approach for the treatment of lung cancer. However, given the challenges posed by the pulmonary route, it is crucial to design carriers capable of circumventing lung clearance mechanisms and ensuring deep drug deposition in the respiratory tract.

From this perspective, CS-based nanoparticles exhibit properties that favor pulmonary delivery and consequently enhance therapeutic efficacy. These properties include mucoadhesive capacity and intrinsic antitumor activity. Numerous studies have demonstrated the successful application of CS-based nanoparticles in inhalation formulations for pulmonary administration, with the aim of treating lung cancer. These studies have demonstrated the feasibility of drying these carriers, their ability to enhance the stability of the encapsulated agent, their potential to increase the drug’s residence time in pulmonary tissue, and their prospect of enhancing antitumor efficacy in in vivo studies.

Furthermore, recent research has demonstrated the feasibility of using CS nanoparticles as an innovative platform for the pulmonary delivery of siRNA, advancing cutting-edge therapy for lung cancer treatment. Despite promising results, some researchers report that one of the disadvantages of using CS nanoparticles is their low transfection efficiency due to insufficient buffering capacity for endosomal escape. However, to overcome this limitation, some studies have suggested grafting chitosan with polyethyleneimine, yielding promising results.

Overall, the primary limitation for the use of CS lies in its limited aqueous solubility, which is pH-dependent. Given the ease of chemical modification of the CS structure, numerous studies have been conducted to synthesize more soluble derivatives of this polymer. Consequently, some authors have demonstrated the feasibility of using N-trimethyl chitosan, glycol chitosan, and carboxymethyl chitosan nanoparticles for lung delivery of antitumor agents

Finally, additional studies are imperative to establish scalable processes for the preparation of CS-based nanoparticles. Moreover, comprehensive preclinical investigations, including those involving inhaler formulations containing CS-based nanoparticles, are essential for the advancement toward clinical trial readiness. 

## Figures and Tables

**Table 1:** Principal Drugs Employed in the Conventional Approach to the Treatment of Lung Cancer.

Drug(s)	Class	Mechanism of Action
Cisplatin Carboplatin	Alkylating agents	Promotes platinum covalent bonds with purine bases, resulting in damage to DNA replication and transcription due to inter- and intra-chain crosslinking [54,55].
Docetaxel Paclitaxel	Taxanes	The alteration of the balance between the formation and degradation of tubulin results in the disruption of the dynamics of microtubules, which in turn affects the process of cell mitosis [56,57].
Erlotinibe	Tyrosine kinase inhibitor	The irreversible inhibition of tyrosine kinase activity in the epidermal growth factor receptor (EGFR) results in impaired autophosphorylation of EGFR-associated tyrosine residues and impaired cell signaling and proliferation [58,59].
Etoposide	Topoisomerase inhibitor	Inhibiting topoisomerase II results in the compromise of the transient breaks in the DNA molecule that occur during cell replication [60,61].
Vincristine	Vinca alkaloids	The compound destabilizes microtubules, which impairs the formation of mitotic spindles. This, in turn, results in the interruption of the cell cycle in the G2/M phase [62]
Erlotinib Gefitinib Afatinib	EGFR-directed tyrosine kinase inhibitors (TKI)	Promotes selective and irreversible inhibition of the epidermal growth factor receptor (EGFR), resulting in inhibition of tumor cell growth and progression [63,64].
Crizotinib Ceritinib Alectinib Lorlatinib	ALK-directed tyrosine kinase inhibitors	Selectively inhibits the ALK tyrosine kinase receptor and its variables, interfering with the survival and proliferation of cancer cells [63,65,66,67].
Ciritinib Lorlatinib Entrectinib	ROS1-directed Therapy	Inhibits the tyrosine kinase ALK and ROS1, reducing the resistance mechanism associated with previous treatment with ALK inhibitor [63,65,68].
Vemurafenib Dabrafenib	BRAF V600E	It selectively inhibits BRAF serine–threonine kinase, interfering with constitutive activation of the RAS/RAF/MEK/ERK signaling pathway and consequently suppressing cell differentiation and proliferation in BRAF V600 mutation-positive tumor cells [63,65,69].
Tepotinib Capmatinib	MET inhibitors	Inhibits the binding of adenosine triphosphate (ATP) to the MET tyrosine kinase receptor, jeopardizing phosphorylation of MET and its downstream effects, thus inhibiting tumor proliferation and inducing apoptosis in MET-dependent tumor cell lines [63,65,70].
Selpercatinib Pralsetinib	RET inhibitors	Selectively inhibits RET kinase small molecules via the ATP-competitive mechanism, compromising the activation of multiple downstream cell signaling pathways, including RAS/MAPK/ERK, PI3K/AKT, and JAK/STAT, and consequently reduces cell proliferation and differentiation [63,65,71].
Sotorasib Adagrasib	KRAS G12C inhibitors	Selectively inhibits the *KRAS G12C* gene (tumor-restricted oncogenic mutant form of KRAS) by interacting with a surface groove of the histidine 95 next to the cysteine 12 switch II pocket, keeping GDP-inactive, compromising oncogenic pathways and uncontrolled cell growth [63,65,72].
T-DXd	HER2-targeted Therapy	Inhibits HER2 overexpression, compromising various proliferative or apoptotic signaling pathways, including MAPK, PI3K/AKT, and JAK/STAT [63,65,73].
Larotrectinib Entrectinib	NTRK inhibitors	Inhibits the kinase portion of the tropomyosin receptor (TRK), thereby impairing cell proliferation [63,65].
Everolimus, Rapamycin Temsirolimus	Immunotherapy and mTOR inhibitors.	It inhibits the proliferation, migration, and survival of cancer cells by forming a complex with the FKBP12 protein, which inhibits the mTOR1 kinase [65,74,75].

Abbreviations: ALK: Anaplastic lymphoma kinase; BRAF: V-Raf murine sarcoma viral oncogene homolog B1; EGFR: Epidermal growth factor receptor; FKBP12: immunophilin 12-kDa protein; HER2: Human epidermal growth factor receptor 2; KRAS: Kirsten Rat Sarcoma viral oncogene homolog; MET: mesenchymal–epithelial transition; NTRK: Neurotrophic tropomyosin receptor kinase; RET: Rearranged during transfection; ROS1: C-ros oncogene 1.

**Table 2:** Overview of the principal methods typically employed to prepare CS nanoparticles.

Methods	Main Characteristics	Advantages	Limitations	Refs.
Covalent crosslinking	Based on covalent crosslinking between the amino group of CS and the aldehyde group of a crosslinking agent, such as glutaraldehyde.	Obtaining nanoparticles of small size and narrow size distribution.	Glutaraldehyde can cause toxicity and compromise the integrity of the drug.	[140,148]
Ionic gelation	The ionic crosslinking occurs in the presence of the protonated amino groups of the CS and the negatively charged groups of the polyanion, such as sodium tripolyphosphate (TPP).	Simplicity, low cost, and potential scalability. Environmentally friendly preparation techniques.	Destabilization of the system when pH changes occur. The production of nanoparticles with a large size (100–400 nm) and a high degree of polydispersity.	[104,143,144,149,150]
Reverse micellar method (microemulsion)	Based on covalent crosslinking between the aqueous phase composed of CS and glutaraldehyde and an organic phase composed of a lipophilic surfactant (cetyltrimethylammonium bromide or sodium 1,4-bis-2-ethylhexylsulfosuccinate) and an organic solvent (n-hexane).	Obtaining nanoparticles with a size of less than 100 nm and with a narrow size distribution.	The utilization of the organic phase and glutaraldehyde has been limited to biomedical applications. The process is lengthy.	[140,150,151]
Coacervation	It results from the colloidal interaction between CS and oppositely charged macromolecules (e.g., sodium alginate) forming insoluble complexes due to solvent repulsion and the consequent separation of the phases.	Simplicity, no organic solvent, mild temperature conditions during processing.	Challenge of precisely controlling the size and size distribution of nanoparticles.	[140,152]
Spray-drying	CS is dissolved in aqueous acetic acid and subsequently mixed with the crosslinking agent. The nanoparticles are formed by passing through a stream of hot air (120 °C to 150 °C).	The procedure is straightforward and does not utilize toxic solvents. Furthermore, it does not necessitate additional separation and drying steps.	Obtaining nanoparticles with a large size. Unsuitable for thermosensitive drugs. Nanoparticle characteristics are highly influenced by operating parameters, including nozzle size, flow rate, and inlet and outlet temperatures.	[104,146,153]

**Table 3:** Chitosan-based pulmonary delivery system for lung cancer treatment.

Drug or siRNA	Justification	Carrier	Evaluation of Nanoparticles	Main Results	Ref.
Paclitaxel or quercetin	Increase drug retention in lung tissue and reduce resistance mechanisms.	Oleic acid-conjugated CS nanoparticles	Analysis of particle-size, zeta potential, and aerodynamic diameter of polymeric microspheres. In vivo pharmacokinetic study and tissue distribution (rats).	Physicochemical properties ideal for lung deposition; increased bioavailability and pulmonary retention of paclitaxel following inhalation administration.	[22]
Resveratrol	Improving therapeutic efficacy in the treatment of lung cancer.	Cationic nanocarrier of CS and lecithin	In vitro anticancer activity (A549 cell line). Drug uptake analysis using flow cytometry; Selectivity index.	Enhanced anticancer activity in lung cells; improved selectivity in human adenocarcinoma cells (A549).	[23]
Silibinin	Optimize lung tissue targeting and antitumor activity.	PLGA/PCL nanoparticles coated with CS	In vitro pulmonary deposition and anticancer activity assay (A549 cell line). In vivo Pharmacokinetic study (rats)	Increased bioavailability and enhanced cell inhibition.	[156]
2-ME	Targeted delivery to the lung and improved clinical efficacy.	PLGA nanoparticles coated with CS nanoparticles	In vitro cytotoxicity and cellular uptake (SPC-A1 and A549 cell lines). In vivo lung deposition and histological examination (rats).	Optimized intracellular drug uptake; improved antitumor activity in A549 cells without promoting an inflammatory response; Promoted deep pulmonary deposition of 2-ME.	[24]
Drug or magnetic nanoparticle	Optimize targeted, non-invasive delivery to lung tissue.	CS nanoparticles	Evaluation of aerodynamic properties; In vitro cellular uptake (A549 cell line) and cell interaction (L929 and A549 cell lines); Determination of drug release profile from nanoparticles.	Physicochemical properties optimized targeted delivery to lung tissue with cellular uptake in A549 cell lines and sustained release profile.	[165]
siRNA-luciferase	Evaluate the biodistribution and gene silencing efficacy of siRNA through the carrier.	CS nanoparticles	In vivo biodistribution of siRNA following pulmonary delivery of the siRNA/chitosan and gene silencing.	Increased retention in lung tissue; effective and specific gene silencing. of metastatic tumor cells in mouse lungs.	[166]
siRNA-EGFP	Optimize lung distribution and gene silencing.	CS nanoparticles	Flow cytometric (H1299 cell line); In vivo Pulmonary deposition assay and RNA Interference (mice).	Delivery system demonstrated safe dosing profile and enhanced gene silencing.	[167]
siRNA-EGFP	Evaluate the efficiency of the siRNA delivery system.	CS nanoparticles	In vivo pulmonary RNA interference in the transgenic EGFP mouse assay.	Enhanced silencing of EGFP expression in vivo in the lower and upper regions of lung tissue.	[168]
a-PDL-1	Enable efficient transmucosal delivery in the treatment of lung metastases.	CS nanoparticles	In vivo transmucosal absorption and permeability assay and in vivo immune responses induced by CS/aPD-L1 nanocomplex assay.	Increased absorption capacity and transmucosal penetration; activation of the immune system resulting in apoptosis of cancer cells.	[155]
Doxorubicin and siRNA-MRP-1	Increase therapeutic efficacy in lung cancer to overcome multidrug resistance.	CS nanoparticles	In vitro anticancer study (H69AR cell line)	Enhanced antiproliferative effect of doxorubicin mediated by multidrug resistance gene silencing.	[169]

CS: Chitosan; PLGA: poly(lactic-co-glycolic) acid; PCL: Polycaprolactone; 2-ME: 2-Methoxyestradiol; EGFP: enhanced green fluorescent protein; a-PDL-1: Programmed-Death Ligand 1.

**Table 4:** Patents attributed to the development of chitosan nanoparticles with pulmonary administration in the treatment of lung.

Patent Name	Patent Number	Country	Chitosan Function	Active Pharmaceutical Ingredient	Ref.
Nano-delivery system for inhaled chemotherapy.	WO2022119528A1	Turkey	Attach the drug to the mucosa of the respiratory system.	Doxorubicin	[203]
Quercetin and paclitaxel co-transportation pulmonary inhaled nanometer targeted porous polymer particle and preparation method thereof.	CN106309411A	China	Providing bioadhesion to the pulmonary mucosa with a sustained release effect; safety by reducing the dosage of conventional chemotherapies and extending the scope of treatment. Provide simultaneous drug delivery.	Quercetin Paclitaxel	[204]
Nanoparticle targeted drug delivery to the lungs using to the extra-testicular Sertoli cells.	WO2009105278A2	United States of America	Drug carrier	Curcumin	[205]
Method for preparing micro nano porous microspheres carrying gene and polypeptide drugs through supercritical fluid technology	CN105963714A	China	Increase the stability of encapsulated agents	siRNA-OD. Polypeptide GLP-1 (glucagon-like peptide-1).	[206]
Composition of tumor-associated proliferative peptides and related anticancer immunogen for the treatment of lung cancers and other cancers.	CN111491660A	China	Immunogen transporter linked to the mimetic peptide.	(A) one or more mimetic peptides, selected from the group consisting of sequences SEQ ID NO 1 to SEQ ID NO 40, consisting of an equal mixture of the following amino acid sequences: CYS-pro-pro-pro -SER-SER-GLN-PRO-LYS-ALA-LEU-GLY-ASN-GLN-GLN-PRO-SER-TRP-ASP-SER-GLU-ASP-SER-SER-ASN-PHE-LYS-ASP(ONKO-5a) (SEQ ID NO: 1) and Cys-pro-pro-pro-pro-SER-SER-TYR-PRO-ARG-GLY-ASN-HIS-TRP-ALA-VAL-GLY-HIS-LEU-MET-NH2 (SEQ ID NO: 9).	[207]
Medicine-carried black phosphorus shell glycan composite nanospheres and preparation method and application thereof.	CN110090307A	China	Chitosan provides the ability to adhere to the lung mucosa and antibacterial capacity.	Not specified	[208]
Methods and compositions for reducing activity of the atrial natriuretic peptide receptor and for treatment of diseases.	CA2707444A1	Canada	Administer the polynucleotide complex intranasally or by nebulization.	siRNA-NPRA (natriuretic peptide receptor A) si-NPRC (natriuretic peptide receptor C)	[209]
Nanopaticles and porous particles anda methods of making the same.	WO2010057214A2	United States of America	Improve the bioavailability of the drug and reduce the frequency of dosing.	Budesonide. Salbutamol sulfate.	[210]
Anticancer drugs, and uses relating for malignant melanoma and other cancers	CN102438449A	India	Increase the bioavailability of silephenol and silephenol triazene.	Selephenoll Selenophenol triazene.	[211]
Dually Derivatized Chitosan Nanoparticles and Methods of Making and Using the Same	US2015051265A1	United States of America	Non-viral nucleic acid delivery.	Therapeutic RNA (antisense RNA, siRNA, short hairpin RNA, micro-RNA, and enzymatic RNA).	[212]
Localized expression of therapeutic nucleic acids in lung epithelial cells	US2023210995A1	United States of America	Nucleic acid transport.	Therapeutic proteins and therapeutic RNA.	[213]

## Data Availability

Data is contained within the article.
